# The importance of factors early in life for development of eating disorders in young people, with some focus on type 1 diabetes

**DOI:** 10.1007/s40519-023-01633-5

**Published:** 2024-01-10

**Authors:** J. Ludvigsson, Å. Olsen Faresjö

**Affiliations:** 1 Crown Princess Victoria Children’s Hospital and Div of Pediatrics, Department of Biomedical and Clinical Sciences, Linköping University, 581 85 Linköping, Sweden; 2 Division of Society and Health/Public Health, Department of Health, Medicine and Caring Sciences, Linköping University, Linköping, Sweden

**Keywords:** Eating disorders, Early life, Type 1 diabetes, Celiac disease, ABIS

## Abstract

**Aim:**

Eating disorders have a serious impact on quality of life, especially when combined with Type 1 diabetes. We investigated eating disorders in relation to factors early in life with some focus on Type 1 diabetes.

**Methods:**

Out of 21,700 children born 1st of Oct 1997–1st of Oct 1999 17,055 (78.6%) were included in ABIS (All Babies in southeast Sweden) and 16,415 had adequate questionnaires. ICD-10 diagnosis from The National Patient Register was merged with the ABIS data.

**Results:**

In total 247 individuals, 19 boys (7.7%) and 219 girls (92.3%) out of 16,415 (1.5%) developed eating disorders (EDs), 167 (1.0%) Type 1 diabetes of whom 7 (4.2%) also got eating disorders (ED) (OR 3.25 (1.47–7.28); *p* = 0.04), all of them years after diagnosis of Type 1 diabetes. EDs was associated with high parental education especially in fathers (OR 1.65 (1.09–2.50); *p* = 0.02) and to at birth anxiety, and depression among mothers. There was no association with the duration of breastfeeding.

**Conclusions:**

Eating disorders are common in girls, with increased risk in high-educated but psychologically vulnerable families. Prevalence is increased in type 1 diabetes. Even modern diabetes treatment needs to be completed with psychological support.

**Level of evidence:**

Level III: Evidence obtained from well-designed cohort or case–control analytic studies.

**Supplementary Information:**

The online version contains supplementary material available at 10.1007/s40519-023-01633-5.

## Introduction

Eating disorders are characterized by a divergent relation to food and eating, or an excessive focus on body weight and weight control [1, 2]. In the general population, eating disorders are associated with extensive co-morbidity and more than 70% of those with eating disorders have an additional psychiatric diagnosis [2]. Most common are mood and anxiety disorders, personality disorders, neurodevelopment disorders, or disorders, including the use of alcohol or drugs [2].

There is a lack of knowledge from longitudinal prospective studies on how early factors in life influence the development of eating disorders in children, beside an increased risk for eating disorders in premature children and with older parents [3]. We, therefore, decided to use the prospective birth cohort ABIS (All Babies in Southeast Sweden) to analyze how psychosocial situation and early lifestyle may contribute to anorexia nervosa and bulimia during the following 20 years of life,

Eating disorders have a serious impact on quality of life for anybody but especially when combined with type 1 diabetes when it causes increased mortality [4]. Thus, one study showed 38% mortality in a group of patients with both type 1 diabetes and anorexia nervosa, which is five times higher mortality than with anorexia nervosa alone [4]. The increased mortality may be caused by weight loss in combination with omitted insulin doses, keto-acidosis, and hyperglycemia [5]. The increased prevalence of eating disorders in adolescents with type 1 diabetes has been estimated to be 6–8% (lifetime prevalence 10.3–14.0%) [6], even though the typical anorexia nervosa, according to some studies, may not be more common in patients with type 1 diabetes than in the general population. Eating disorders have usually been regarded as a consequence of having type 1 diabetes, decreasing quality of life [6]. With more restrictive dietary rules for patients with type 1 diabetes eating disorders were supposed to be a quite common consequence decades ago, but one would expect that this complication has become rare as modern treatment allows a less strict diet. However, the prevalence of eating disorders may still be high as intense insulin treatment is based on carbohydrate counting, in practice “diet”, and food habits are still an important part of type 1 diabetes treatment. Furthermore, there are studies suggesting that the association between eating disorders and autoimmune diseases may be complex, and that eating disorders actually may forego the diagnosis of autoimmune diseases, and in some way contribute to the development of, e.g., type 1 diabetes and not only be a consequence of dietary treatment [7]. Previous studies of eating disorders in type 1 diabetes are mainly based on cross-sectional associations. In this prospective study, we elucidate this question further by also investigating when eating disorders develop in patients with celiac disease, another autoimmune disease with dietary rules as an important part of treatment.

The aim of our study was, therefore, primarily to analyze how psychosocial factors in early life may contribute to eating disorders, especially anorexia nervosa, during the following 20 years of life, but secondary also to investigate the association between these disorders and type 1 diabetes or celiac disease.

## Materials and methods

Our data derive from the longitudinal prospective population-based birth cohort study ABIS (All Babies in Southeast Sweden). All 21,700 mothers who gave birth to a child between 1st of October 1997 and the 1st of October 1999 were asked to participate in the ABIS study and 17 055 (78.6) did so, giving a study population which has been shown to be well-representative of the general Swedish population. ABIS was designed to identify environmental and genetic factors associated with development of immune-mediated, especially type 1 diabetes and other autoimmune diseases. Questionnaires were answered by parents at the birth of their child and then 1, 2.5–3, and 5 years later. At 8 and at 10–12 years, separate questionnaires were answered by both children and parents, and at 17–19 years by the teenagers. At every follow-up, several biological samples were collected. In total, 16,415 ABIS individuals could be included in this study with adequate questionnaires. National register data based on medical records and ICD-10 diagnosis from The Swedish National Board of Health and Welfare were merged with the ABIS data. The diagnoses of anorexia nervosa (F50.0, F50.8, F50.9), Bulimia (F50.2) type 1 diabetes (E10), celiac disease (K90), depression (F39.1) and anxiety (F42.1) were collected from the national register.

The control group consists of 16,168 participants in the study without any of the four F50.0, F50.8, F50.9 and F50.2 diagnoses.

The following variables were used derived from the questionnaires at birth: age of mothers at birth, educational level, family composition, and number of siblings. From the questionnaire answered at 1 year of age we included duration of breastfeeding, which might influence both contacts between mother and child, and influence the gut microbiome, and from the questionnaire at 2.5–3 years, we included early symptoms of dysbiosis (stomach ache or vomiting). Household income of the family is based on information from year 2000, in terms of the family income available after taxation, collected from Statistics Sweden and divided into the quintiles low, quite low, middle, quite high, and high. Further description of variables from the questionnaires is given in Appendix.

## Statistics

Statistical analyses were made in IBM SPSS Statistics 28.0. Chi-squared test was used to compare the variables for the cases and controls. Multiple multivariate binary regressions were performed to assess the relationship and risks (OR, 95% CI) between the dependent variable, eating disorder compared to the control group. Variables for multivariate binary regression analyses were selected based on significant values in univariate analyses using Chi-squared and correlations tests. A *p* value less than 0.05 was statistically significant.

## Results

In total 247 individuals, 19 boys (7.7%) and 219 girls (92.3%) out of 16,415 (1.5%) developed eating disorders (probands), 167 (1.0%) developed type 1 diabetes of whom 7 (4.2%) also eating disorders (OR 3.25 (1.47–7.28); *p* = 0.04) and 228 (1.4%) developed celiac disease of whom 6 (2.6%) also eating disorders. Parental educational level was significantly higher in probands, especially so in the fathers (Table 1). Mothers of probands reported already at birth of the probands health problems such as anxiety, and depression but also type 2 diabetes more frequently than the control group. There was no association with the duration of breastfeeding, but early symptoms of vomiting were more frequent among the probands. There was no association between development of eating disorders and BMI of their parents at repeated follow-ups (1, 2.5–3, 5 and 8 years of age).

**Table 1:** Characteristics and early symptoms for cases and controls

Variable	Cases (247) % (*n*)	Controls (16,168) % (*n*)	*p*-value*
Gender			< 0.001
Boys	7.7 (19)	52.5 (8465)	
Girls	92.0 (228)	47.5 (7652)	
Maternal age at birth			0.19
15–19 years	1.3 (3)	1.4 (223)	
19–25 years	16.9 (40)	21.0 (3311)	
26–35 years	69.6 (165)	68.6 (10,788)	
36–47 years	12.2 (29)	9.0 (1415)	
Maternal education level			0.02
Low	8.8 (21)	8.6 (1357)	
Middle	52.5 (126)	59.7 (9399)	
High	20.0 (48)	19.3 (3038)	
Extra high	18.8 (45)	12.3 (1938)	
Paternal education level			0.002
Low	10.2 (24)	13.7 (2118)	
Middle	56.2 (132)	61.9 (9594)	
High	13.6 (32)	12.2 (1897)	
Extra high	20.0 (47)	12.2 (1889)	
Family situation at birth			0.23
Single	1.7 (4)	2.1 (336)	
Couple	50.6 (122)	55.6 (8758)	
Married	47.7 (115)	42.3 (6668)	
Number of siblings			0.38
No siblings	40.5 (100)	39.5 (63,919	
1–2	54.7 (135)	52.8 (8535)	
3–4	4.5 (11)	6.5 (1047)	
5–9 or more	0.4 (1)	1.2 (195)	
Household income			0.16
Low	15.0 (37)	20.1 (3221)	
Quite low	21.0 (52)	20.0 (3199)	
Middle	18.2 (45)	20.0 (3205)	
Quite high	24.7 (61)	19.9 (3190)	
High	21.1 (52)	19.9 (3191)	
Breastfeeding (exclusive)			0.41
0–3 months	18.2 (31)	23.0 (2615)	
4–6 months	71.8 (122)	67.1 (7613)	
7–8 months	5.3 (9)	6.2 (700)	
9 or more	4.7 (8)	3.7 8421)	
Breastfeeding (total)			0.27
0–3 months	8.9 (14)	12.3 (1355)	
4–6 months	16.5 (26)	18.9 (2081)	
7–8 months	27.7 (39)	25.8 (2841)	
9 or more	50.0 (79)	42.9 (4720)	
The mother reported bad health at the time for child delivery			0.03
Yes	5.0 (12)	2.7 (47)	
No	95.0 (227)	97.3 15,177)	
Living situation of the mother During pregnancy			0.11
Community	28.4 (67)	31.8 (4916)	
Countryside	11.0 (26)	14.2 (2200)	
Town	60.6 (143)	54.0 (8339)	
Stomach ache in children^a^			0.46
Yes	7.3 (9)	5.8 (491)	
No	92.7 (114)	94.2 (8037)	
Vomiting in children^a^			0.008
Yes	2.2 (3)	0.6 (49)	
No	97.8 (123)	99.4 (8623)	

^a^Data collected at 2.5 year follow-up

Comorbidities of probands are shown in Table 2. Compared to the controls, the probands had more often also been diagnosed with anxiety (17.2% versus 2.7%) (*p* < 0.001), depression ((10.1% versus 2.4%) (*p* < 0.001), and type 1 diabetes (2.9% versus 0.99%) (*p* = 0.004).

**Table 2:** Comorbidities, the diagnose prevalence among cases and controls

Comorbidity	Cases (*n* = 247) % (*n*)	Controls (*n* = 16,177) % (*n*)	*p*-value
Anxiety	16.6 (41)	2.7 (443)	< 0.001
Depression	10.5 (26)	2.4 (383)	< 0.001
Diabetes type 1	2.8 (7)	1,0 (160)	0.004
Celiac disease	2.4 (6)	1.4 (222)	0.16

**Fig. 1:**
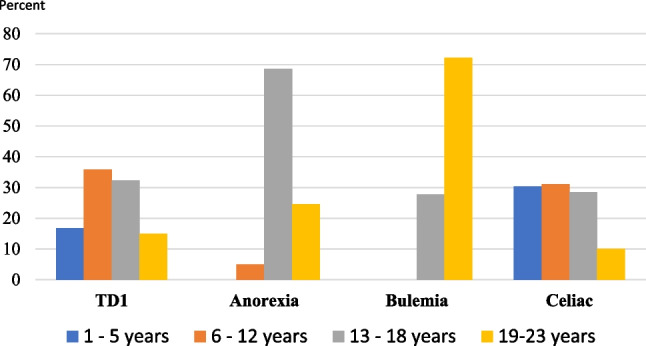
Distribution ages of disease debut concerning Type 1 diabetes, anorexia nervosa, bulimia, and celiac among the ABIS study population

Age at diagnosis of type 1 diabetes, anorexia nervosa, bulimia, and celiac disease is shown in Fig. 1. A majority (62.5%) of individuals with both depression and eating disorders got their two diagnoses the same year and half of the cases with anxiety (51.2%) and eating disorders. However, all type 1 diabetes individuals got their diagnosis of anorexia nervosa or bulimia years after they had got the type 1 diabetes diagnosis. Similarly, in individuals with both celiac disease and eating disorder; all the patients got the celiac disease diagnosis years before the eating disorder.

Multiple logistic regression of factors associated with eating disorder diagnoses is shown in Table 3. Thus, it is evident that high paternal education as well as factors such as depression and anxiety in the children with ED remain significantly related to the risk of developing eating disorders.

**Table 3:** Multiple logistic regression of factors associated with eating disorders diagnoses

*Variabel*	*p*-value	OR (95% CI)
Gender	*p*-value	OR 95% confidence interval
Boys	*Reference*	*Reference*
Girls	< 0.001	11.22 (6.99–18.00)
Paternal education level		
Medium (High school)	*Reference*	*Reference*
Low (Elementary school)	0.66	0.90 (0.57–1.42)
High (1–3 year university)	0.43	1.18 (0.78–1.79)
Extra high (> 3 year university)	0.02	1.65 (1.09–2.50)
Maternal education level		
Middle (high school)	*Reference*	*Reference*
Low (elementary school)	0.75	1.09 (0.65–1.84)
High (1–3 year university)	0.68	1.08 (0.75–1.55)
Extra high (> 3 year university)	0.16	1.35 (0.90–2.06)
Mother reported bad health at birth		
No	*Reference*	*Reference*
Yes	0.001	2.25 (1.26–4.14)
Anxiety		
No	*Reference*	*Reference*
Yes	< .001	4.24 (2.85–6.31)
Depression		
No	*Reference*	*Reference*
Yes	< .001	2.63 (1.62–4.26)
Type 1 diabetes		
No	*Reference*	*Reference*
Yes	0.004	3.25 (1.47–7.28)

Logistic regression model: df = 15, *p*-value ≤ 0.001, Cox and Snell R square = 0.020, Nagelkerke R square = 0.137

## Discussion

In the general population, eating disorders are rather common, mainly in girls. We find early life risk factors in addition to earlier results [3]. Already at birth of the child, mothers of children who later develop eating disorders (the probands) more often had reported anxiety and/or depression, which may indicate a more psychologically vulnerable family. One can speculate that the increased risk of eating disorders in children of parents with high education, in agreement with earlier findings of connection between anorexia and higher socioeconomic status [8]**,** is related to higher expectations which may become an extra burden for the child. That vomiting was reported more often already at 3 years of age in the probands may suggest a disposition to gastrointestinal problems.

Comorbidity with anxiety and depression underlines that eating disorders are part of broader psychological problems, and even the most modern treatment of Type 1 diabetes needs to be completed with psychological support not least in high-educated families with history of earlier psychological problems.

Still with modern quite successful diabetes treatment, eating disorders are more common in individuals with type 1 diabetes, and somewhat more common also in patients with celiac disease. It has been suggested that there is a non-genetic association between eating disorders and autoimmune diseases, especially type 1 diabetes, with increased prevalence of eating disorders already before diagnosis of type 1 diabetes [7], but in our study, there is no such indication. All individuals with eating disorders got their diagnosis years after their diagnosis of type 1 diabetes resp celiac disease, suggesting that the eating disorders in young individuals are a consequence of having type 1 diabetes and/or celiac disease. In type 1 diabetes, it is well-known that the patients, usually teenagers or young adults sometimes deliberately omit insulin to lose weight, or restrict their eating, or alternate between vomiting and binge eating. That eating disorders also are quite common in individuals some years after the diagnosis of celiac disease suggests that the dietary rules may contribute to eating disorders, even if it may be so that people who get their celiac disease diagnosed in adulthood may have had problems with food intake and eating disorders before their celiac disease was discovered [9]. Connections between anorexia nervosa and autoimmune/inflammatory disorders, e.g., inflammatory bowel disease may be explained by similar mechanisms [10].

### Strength and limitations

This study has several strengths with the prospective follow-up from birth of a large general population and can therefore give reliable information on the importance of early factors in life for the development of eating disorders. However, there are limitations with regard to development of eating disorders in type 1 diabetes, as the numbers inevitably are small with only 7 cases with that combination in a population of 17,000 individuals. However, as all cases got their eating disorder several years after their diagnosis of type 1 diabetes, that picture seems rather robust. Another limitation could be use of only the birth questionnaire concerning demographic variables, which might have changed during the follow-ups. However, the focus of our study was to study the importance of factors early in life.

## Conclusions

Factors early in life may contribute to development of eating disorders many years later. Anorexia nervosa and Bulimia are especially common in individuals with type 1 diabetes, with onset several years after type 1 diabetes is diagnosed, suggesting that type 1 diabetes and its treatment causes this dangerous combination. Celiac disease shows a similar relationship. Even modern diabetes treatment needs to be completed with psychological support, not least in high-educated families with history of earlier psychological problems.

## What is already known on this subject?

Associations to socioeconomic status and development of eating disorders are shown earlier, but there is lack of knowledge about the importance of factors early in life from prospective studies with long-term follow-up. That type 1 diabetes is associated with development of eating disorders is known.

## What this study adds?

Already at birth of the child mothers of children who later develop eating disorders more often had reported anxiety and/or depression, and there is an increased risk of eating disorders in children of parents with high education. Still with modern treatment of type 1 diabetes prevention of eating disorders needs early psychological support, especially in families with high educated parents with previous psychological symptoms.

### Supplementary Information

Below is the link to the electronic supplementary material.

Supplementary file1 (DOCX 24 KB)

## Data Availability

The data sets generated during and/or analyzed during the current study are available from the corresponding author on reasonable request.

## Abbreviations

EDs: Eating Disorders
T1D: Type 1 diabetes
HbA1c: Haemoglobin A1c
OR: Odds Ratio
ABIS: All Babies in Southeast Sweden
