# Supplementary material for: The importance of factors early in life for development of eating disorders in young people, with some focus on type 1 diabetes
Source: Eat Weight Disord. 2024 Jan 10;29(1):5. doi: 10.1007/s40519-023-01633-5 (PMC10781866; doi:10.1007/s40519-023-01633-5)
Supplement: Supplementary file 1 — Supplementary file1 (DOCX 24 KB) [file 40519_2023_1633_MOESM1_ESM.docx]

# APPENDIX 1

Correlations analysis of all variables

| Variabel | p-value* | r-value* |
| --- | --- | --- |
| Baseline characteristics |  |  |
| Gender | <0.001 | 0.11 |
| Maternal age at birth | 0.05 | 0.02 |
| Maternal education level | 0.03 | 0.02 |
| Paternal education level | <0.001 | 0.03 |
| Family situation | 0.07 | 0.02 |
| Number of siblings | 0.12 | -0.02 |
| Household income | 0.01 | 0.01 |
| Risk factors during pregnancy and birth |  |  |
| Living situation of the mother during pregnancy | 0.01 | 0.020 |
| Mother reported bad health at birth | 0.02 | 0.02 |
| Risk factors during childhood |  |  |
| Breastfeeding ended | 0.04 | 0.02 |
| Suffer from/affected by poor appetite at 2.5 years | 0.04 | -0.02 |
| Comorbidities |  |  |
| Anxiety | <0.01 | 0.10 |
| Depression | <0.001 | 0.59 |
| Diabetes type 1 | 0.003 | 0.02 |
| Celiac disease | <0.001 | 0.10 |

*Spearman rho

Binary logistic regression analysis of baseline characteristics

| Variable | Significance | OR (95% CI) |
| --- | --- | --- |
| Gender |  |  |
| Boys | *Reference* | *Reference* |
| Girls | < 0.001 | 11.95 (7.37-19.39) |
| Maternal age at birth |  |  |
| 26–35 years | *Reference* | *Reference* |
| 15-19 years | 0.75 | 0.75 (0.18-3.16) |
| 19-25 years | 0.40 | 0.85 (0.59-1.24) |
| 36-47 years | 0.32 | 1.24 (0.81-1.92) |
| Maternal education level |  |  |
| Middle | *Reference* | *Reference* |
| Low | 0.41 | 1.25 (0.73-2.13) |
| High | 0.99 | 1.00 (0.69-1.45) |
| Extra high | 0.37 | 1.22 (0.79-1.88) |
| Paternal education level |  |  |
| Middle | *Reference* | *Reference* |
| Low | 0.32 | 0.78 (0.49-1.27) |
| High | 0.68 | 1.10 (0.71-1.68) |
| Extra high | 0.02 | 1.65 (1.08-2.52) |
| Household income |  |  |
| Middle | *Reference* | *Reference* |
| Low | 0.12 | 0.673 (0.41-1.11) |
| Quite low | 0.92 | 1.021 (0.66-1.58) |
| Quite high | 0.34 | 1.221 (0.81-1.83) |
| High | 0.49 | 0.857 (0.55-1.33) |

Binary logistic regression analysis of potential risk factors during pregnancy and birth

| Variable | Significance | OR (95% CI) |
| --- | --- | --- |
| Living situation of the mother during pregnancy |  |  |
| Community | *Reference* | *Reference* |
| Countryside | 0.65 | 0.90 (0.56-1.44) |
| Town | 0.04 | 1.38 (1.02-1.87) |
| Mother reported bad health at birth |  |  |
| No | *Reference* | *Reference* |
| Yes | 0.04 | 1.88 (1.02-3.48) |

Logistic regression model: df=3, p-value=<0.001, Cox and Snell R square=0.001, Nagelkerke R square=0.005

Binary logistic regression analysis of potential risk factors during childhood

| Variable | Significance | OR (CI 95%) |
| --- | --- | --- |
| Breastfeeding ended |  |  |
| 7-8 months | *Reference* | *Reference* |
| 0-3 months | 0.50 | 1.52 (0.45-5.15) |
| 4-6 months | 0.84 | 0.88 (0.26-2.96) |
| ≥9 months | 0.76 | 1.14 (0.49-2.65) |
| Suffer from/affected by poor appetite at 2.5 years |  |  |
| No | *Reference* | *Reference* |
| Yes | 0.49 | 1.53 (0.45-5.17) |

– Binary logistic regression of comorbidities

| Variabel | Significance | OR (CI 95%) |
| --- | --- | --- |
| Anxiety |  |  |
| *No* | *Reference* | *Reference* |
| *Yes* | < 0.001 | 5.94 (4.06-8.68) |
| Depression |  |  |
| *No* | *Reference* | *Reference* |
| *Yes* | < 0.001 | 2.46 (1.523-3.98) |
| Diabetes type 1 |  |  |
| *No* | *Reference* | *Reference* |
| *Yes* | 0.02 | 2.56 (1.16-5.65) |
| Celiac |  |  |
| *No* | *Reference* | *Reference* |
| *Yes* | 0.13 | 1.81 (0.83-3.95) |

Logistic regression model: df=4, p-value=<0.001, Cox and Snell R square=0.006, Nagelkerke R square=0.045
